# Molecular insights into the improved clinical performance of PEGylated interferon therapeutics: a molecular dynamics perspective†

**DOI:** 10.1039/c7ra12480e

**Published:** 2018-01-09

**Authors:** Dong Xu, Nikolai Smolin, Rance K. Shaw, Samuel R. Battey, Aoxiang Tao, Yuying Huang, Shaikh Emdadur Rahman, Matthew L. Caylor

**Affiliations:** Department of Biomedical and Pharmaceutical Sciences, College of Pharmacy, Kasiska Division of Health Sciences, Idaho State University Meridian ID 83642 USA xudong@isu.edu +1-208-373-1832; Department of Cell and Molecular Physiology, Loyola University Chicago Maywood IL 60153 USA; The Reynolds Law Firm, P.C. 225 SW 4th St Corvallis OR 97333 USA; Department of Chemistry, Washington State University Pullman WA 99164 USA

## Abstract

PEGylation is a widely adopted process to covalently attach a polyethylene glycol (PEG) polymer to a protein drug for the purpose of optimizing drug clinical performance. While the outcomes of PEGylation in imparting pharmacological advantages have been examined through experimental studies, the underlying molecular mechanisms remain poorly understood. Using interferon (IFN) as a representative model system, we carried out comparative molecular dynamics (MD) simulations of free PEG*x*, apo-IFN, and PEG*x*-IFN (*x* = 50, 100, 200, 300) to characterize the molecular-level changes in IFN introduced by PEGylation. The simulations yielded molecular evidence directly linked to the improved protein stability, bioavailability, retention time, as well as the decrease in protein bioactivity with PEG conjugates. Our results indicate that there is a tradeoff between the benefits and costs of PEGylation. The optimal PEG chain length used in PEGylation needs to strike a good balance among the competing factors and maximizes the overall therapeutic efficacy of the protein drug. We anticipate the study will have a broad implication for protein drug design and development, and provide a unique computational approach in the context of optimizing PEGylated protein drug conjugates.

## Abstract 2

We discovered molecular evidence that links PEGylation to improved clinical performance, yet at the expense of decreased bioactivity. Our computational approach will facilitate PEGylated protein drug design and optimize its overall therapeutic efficacy.

## Graphical abstract

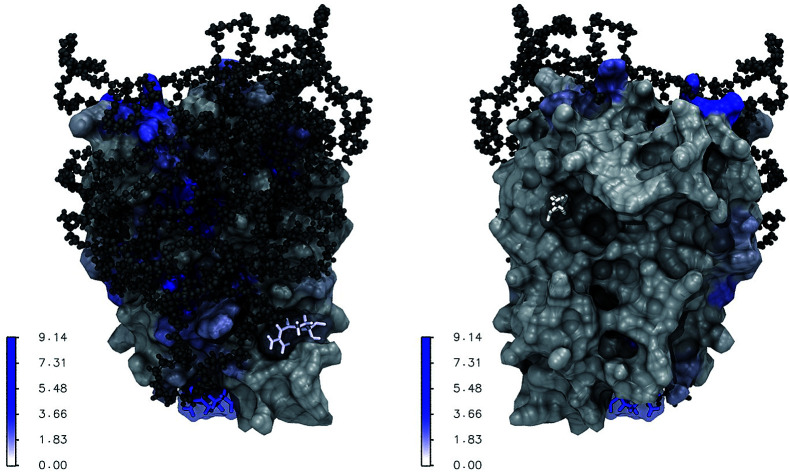

## 1. Introduction

**Fig. 1:**
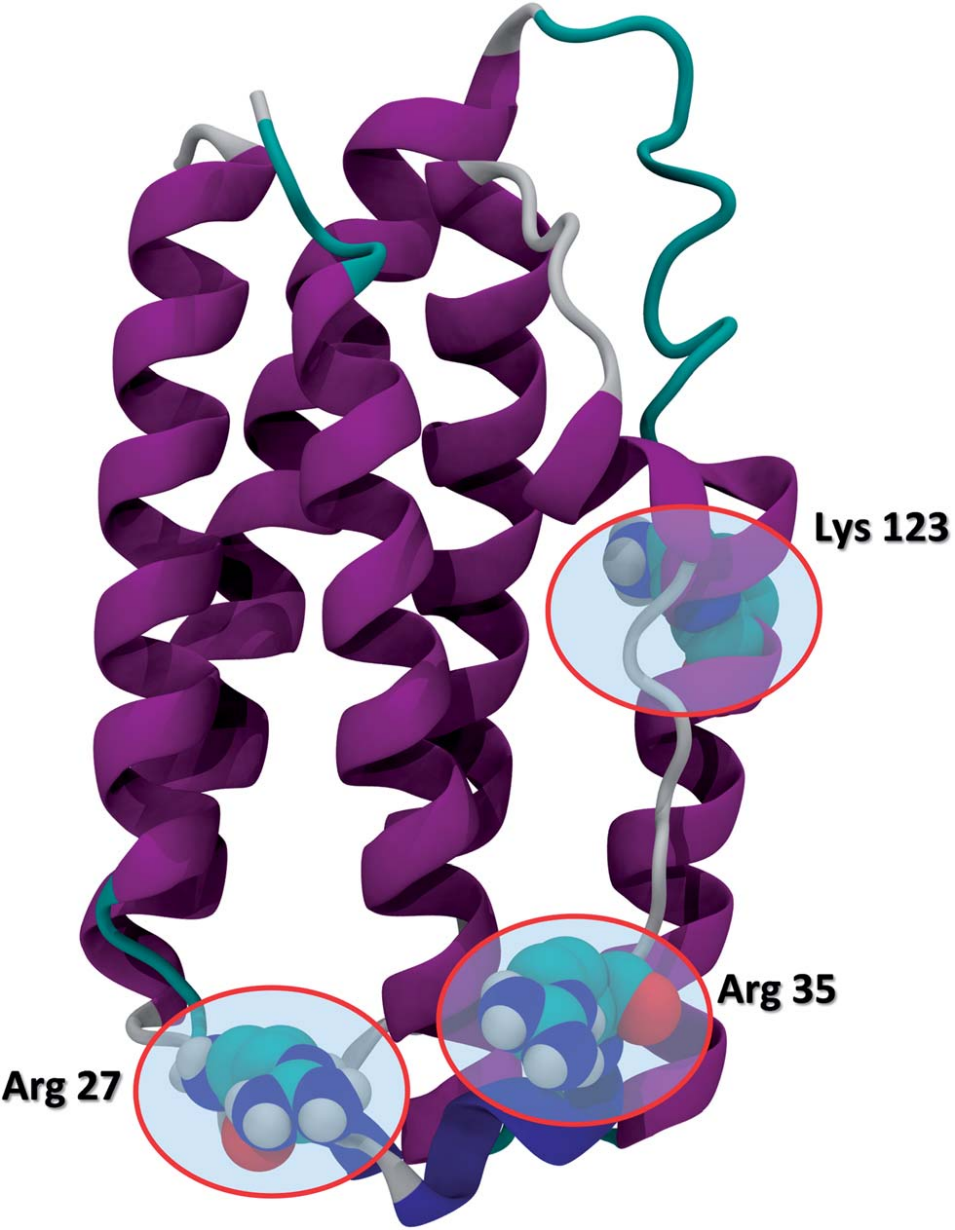
The secondary structure of IFN (PDB ID: 1AU1). IFN is shown in the cartoon representation. Key residues Arg 27, Arg 35, and Lys 123 relevant to IFN antiviral activity are highlighted in the CPK representation.

Recent advances in recombinant DNA technology have led to a significant increase of protein therapeutics development and production. Interferons (IFNs) are a representative class of immunomodulatory protein drugs. IFNs are an evolutionarily conserved family of signaling cytokines that participate as extracellular messengers in a wide range of host responses.^1^ IFNs are 166-residue proteins comprised of 5 alpha helices and weighs about 20 kDa. The secondary structure of the protein and the key residues Arg 27, Arg 35, and Lys 123 known to be crucial to IFN antiviral activity are shown in Fig. 1.^2^ Binding of IFNs to specific cell surface receptors triggers an intracellular signaling cascade resulting in the synthesis of proteins that mediate immunomodulatory responses to maintain homoeostasis and host defense.^3^ IFNs are the first-line treatment of multiple sclerosis^4^ and also have been shown to possess antiviral activity^5,6^ and anti-proliferative effects on tumor cells.^7^

IFNs and many other protein therapeutics suffer from bioavailability issues such as immunogenicity, protein aggregation, rapid renal clearance, and instability, leading to a shortened serum half-life and reduced therapeutic efficacy. As a results, frequent parenteral administrations are necessary, which significantly increase medication costs and skin adverse reactions in patients. Immunogenic effects are observed when a self-antigen is offered to the immune system in the same manner over an extended period of time. The immune system's tolerance for the protein deteriorates, and the protein is targeted for elimination.^8^ Aggregation is commonplace in protein drugs as a result of insolubility. Aggregation is an entropically favorable process that minimizes the exposure of protein hydrophobic surface residues by driving the hydrophobic residues of one protein in contact with the hydrophobic residues of another protein. Protein instability can be attributed to the same entropic effects because proteins have a tendency to fold into conformations in which the number of solvent-exposed surface residues is minimized.^9^ Rapid renal clearance is the result of a combination of IFN's small size and its identification as a xenobiotic.

PEGylation, a process that covalently attaches polyethylene glycol (PEG) polymer chains to macromolecules, is a widely adopted strategy to alter the pharmacokinetic and pharmacodynamic properties of protein therapeutics such as IFNs. The benefits of PEGylation can be achieved by a number of mechanisms, including increased protein stability, solubility, and bioavailability, reduced renal clearance, degradation by proteolysis, and antibody-mediated clearance.^10–14^ In addition, the hydrophobic nature of IFNs makes them prone to protein aggregation *in vivo*, an issue that can be effectively addressed by PEGylation.^15^ Nevertheless, an important requirement is that PEGylation must enhance the pharmacokinetic properties of the protein without significantly affecting its therapeutic activity.

Non-PEGylated IFNs have been experimentally characterized using Fourier transform infrared spectroscopy, differential scanning calorimetry, and hydrogen–deuterium exchange mass spectroscopy.^16,17^ There has been only a limited number of simulations studies that involved PEGylated biosystems.^18–23^ The molecular mechanisms underlying the effects of PEGylation on protein therapeutics, however, are still poorly understood.

Here we present a comparative molecular dynamics (MD) study that specifically examines the molecular mechanisms of how PEGylation affects IFN molecular structures and therapeutic properties with respect to different PEG chain lengths. We further analyze the molecular interactions between PEG and IFN, and reveal their implications to the benefits and costs of PEGylation. Our goal of this study is to provide atomic insights into the effects of PEGylation on IFN with the expectation that they will be used to better understand the mechanisms of PEGylation in a broader range of protein therapeutics.

## 2. Results and discussion

### 2.1 Site of PEGylation

PEG is not reactive to covalently bond to a protein by itself, so a linker molecule must be used. Studies have shown that reductive amination by the PEG-propionaldehyde linker, a widely adopted PEGylation process, selectively PEGylates the primary amine of protein N-terminus.^2^ While there are other potential PEGylation sites, they are less common. Therefore, we focused on PEGylations at the protein N-terminus in this study.

### 2.2 MD simulation validation

A small number of simulation studies have been published for various PEGylated biosystems.^18–23^ These earlier studies, however, did not parametrize the PEGs in a systematic fashion, nor did they validate these PEG models and MD simulations against experimental data. Here we used the persistence length (*λ*), a fundamental mechanical property quantifying the stiffness of a polymer^24^ and one of the most important metrics, to evaluate the quality of our MD simulations involving PEGs. To check if the MD simulations were capable of reproducing the experimentally observed mechanical property for the PEGs, the mean end-to-end distances *h*^2^ were calculated from the MD simulations (see Materials and methods section). Nonlinear fitting of eqn (1) yielded the persistence length *λ* = 3.67 ± 0.14 Å, consistent with the experimental measurements obtained from atomic force microscopy^25^ and other techniques^26^ (Table 1). The excellent agreement between the calculated and experimental data supports that the MD simulations based on our AM1-BCC partial charge model accurately reproduced the correct characteristics and behavior of free and conjugated PEGs.

**Table 1:** PEG persistence length (*λ*)

Experimental^25^	3.80 ± 0.02 Å
Experimental^26^	3.73 ± 0.29 Å
Calculated (this work)	3.67 ± 0.14 Å

### 2.3 Effects of PEGylation on IFN stability

**Fig. 2:**
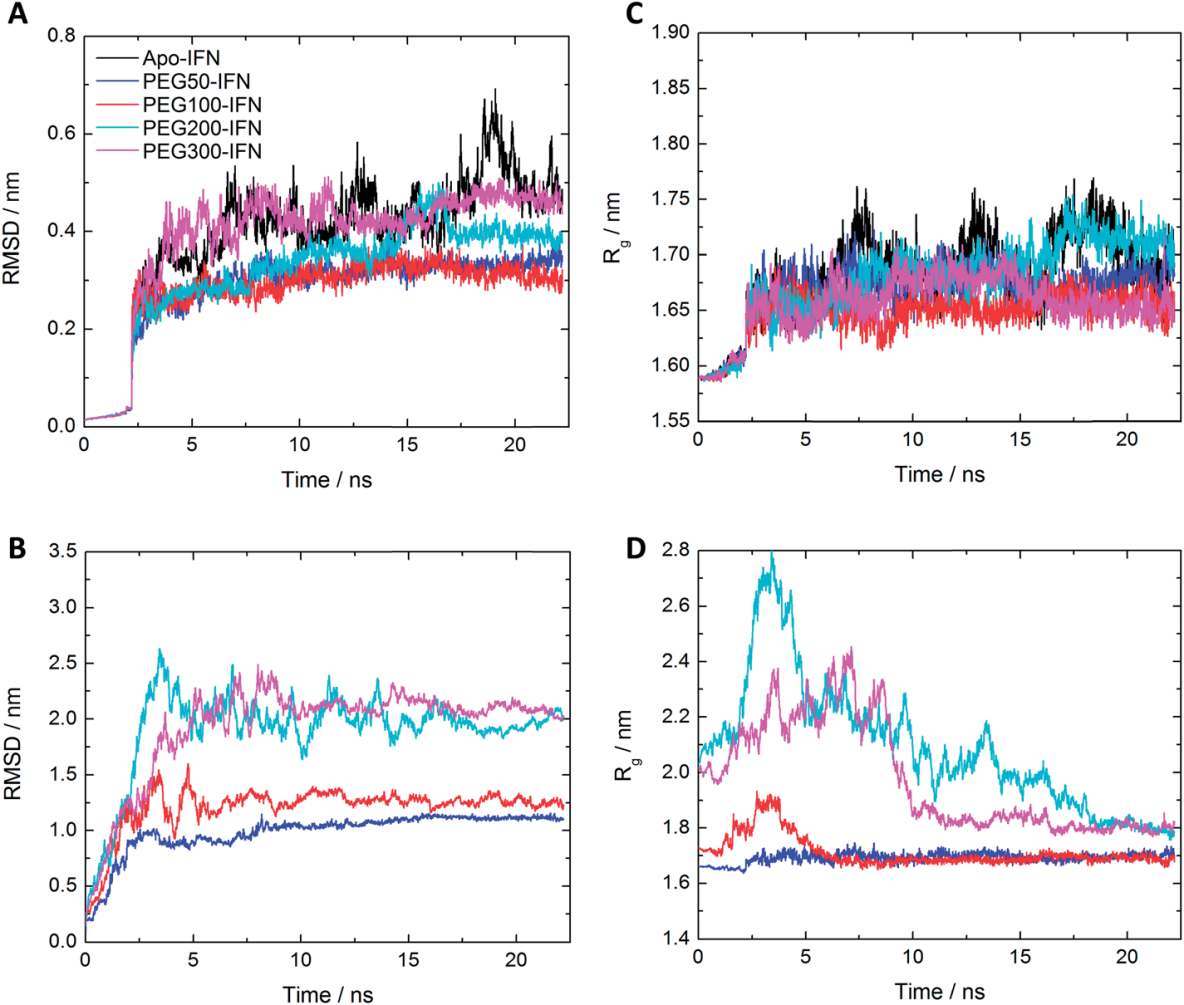
(A) Protein-only RMSD of apo-IFN and conjugated PEG*x*-IFNs; (B) overall RMSD (protein and PEG) of conjugated PEG*x*-IFNs; (C) protein-only *R*_g_ of apo-IFN and conjugated PEG*x*-IFNs; (D) overall *R*_g_ (protein and PEG) of conjugated PEG*x*-IFNs.

The root mean square deviation (RMSD) of Cα atoms of the apo-IFN and PEG*x*-IFNs (protein only) as a function of the simulation time is shown in Fig. 2A. The IFN crystal structure was used as a reference structure for superimposing the MD trajectories in the RMSD calculations. The RMSD of apo-IFN fluctuates around 0.45 nm whereas the RMSDs for the PEG*x*-IFNs are comparatively smaller, indicating a stabilizing effect of the attached PEG chain on the IFN conformation. IFN protein RMSD was only slightly increased with the increase of PEG chain length. In Fig. 2B, the RMSD of the PEG*x*-IFN (protein and PEG) showed relatively large fluctuations compared to the protein-only RMSDs in Fig. 2A due to the movement of the flexible PEG chains included in the calculations. However, the fluctuations quickly plateaued after 5 ns into the simulations. The results showed it only took approximately 5 to 15 ns for the attached PEG chain to fold into low-energy configurations on the IFN surface.

The change of radii of gyration (*R*_g_) over time presented a similar picture. Fig. 2C shows that the IFNs in PEG*x*-IFNs became more compact than the apo-IFN. Fig. 2D shows that the overall size of the PEG*x*-IFN conjugated systems (protein and PEG) stabilized as the PEG chains adopted low-energy configurations on the IFN surface. Similar to the RMSD data, the longer the PEG chain length, the longer the time required to fold into low-energy configurations. The final overall size of the PEG*x*-IFN conjugated systems (protein and PEG) is only marginally larger than the size of the apo protein. The mean *R*_g_ increased from 1.7 nm in apo-IFN to 1.8–1.9 nm in PEG200-IFN and PEG300-IFN. The final overall sizes of PEG100-IFN and PEG50-IFN were almost identical to that of the apo IFN, indicating tight wrapping of the PEG chains on the IFN surface.

It is worth noting that the overall RMSD and *R*_g_ data (Fig. 2B and D) showed that all the simulations had indeed converged at 25 ns. The small fluctuations seen in the apo-INF protein (Fig. 2A and C) corroborates our aforementioned perspective that PEGylation stabilizes INF by holding the protein tightly together. Without PEGylation, INF would be more conformationally flexible due to the lack of β sheets.

**Fig. 3:**
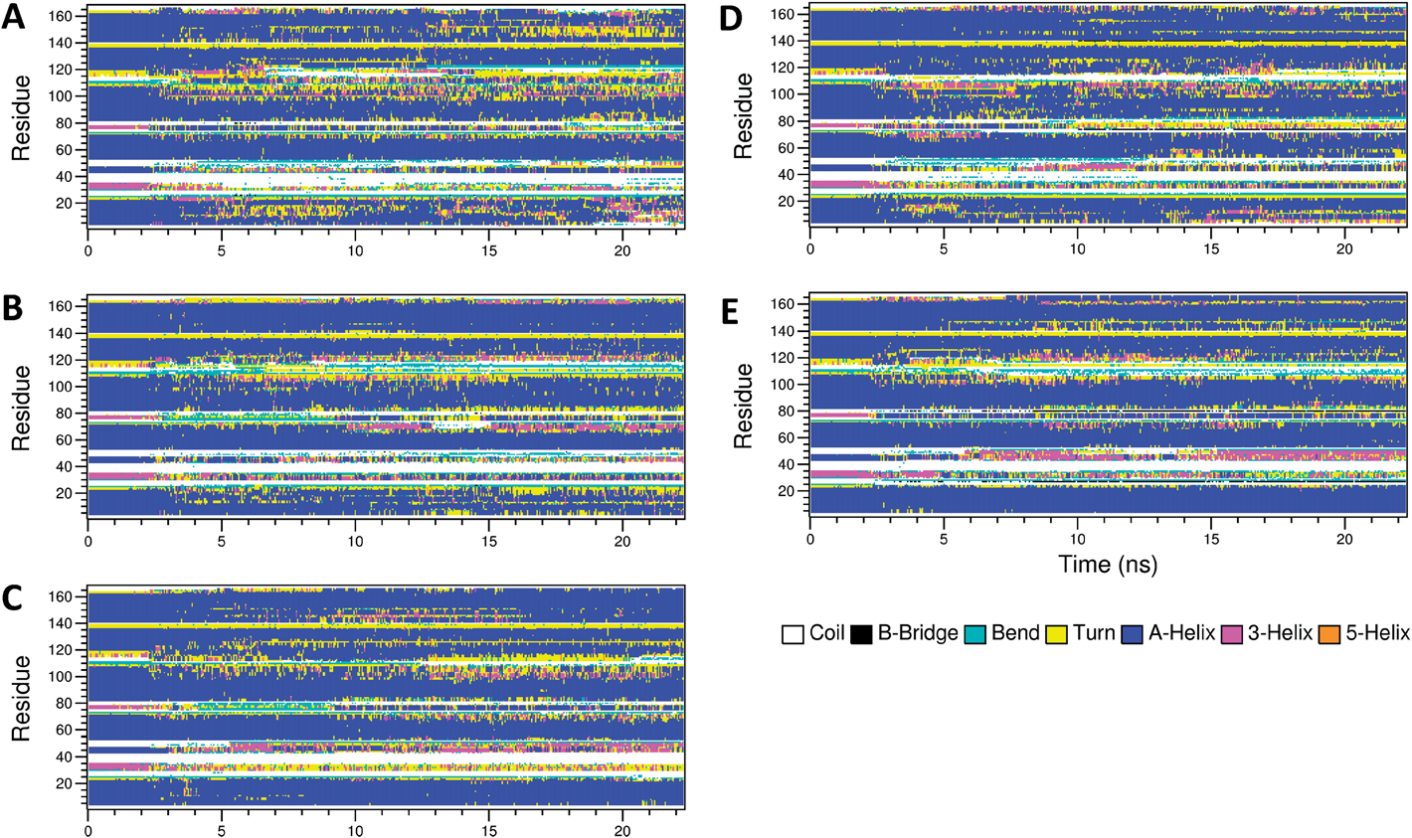
Secondary structures of apo-IFN and conjugated PEG*x*-IFNs. (A) Apo-IFN; (B) PEG50-IFN; (C) PEG100-IFN; (D) PEG200-IFN; (E) PEG300-IFN.

**Fig. 4:**
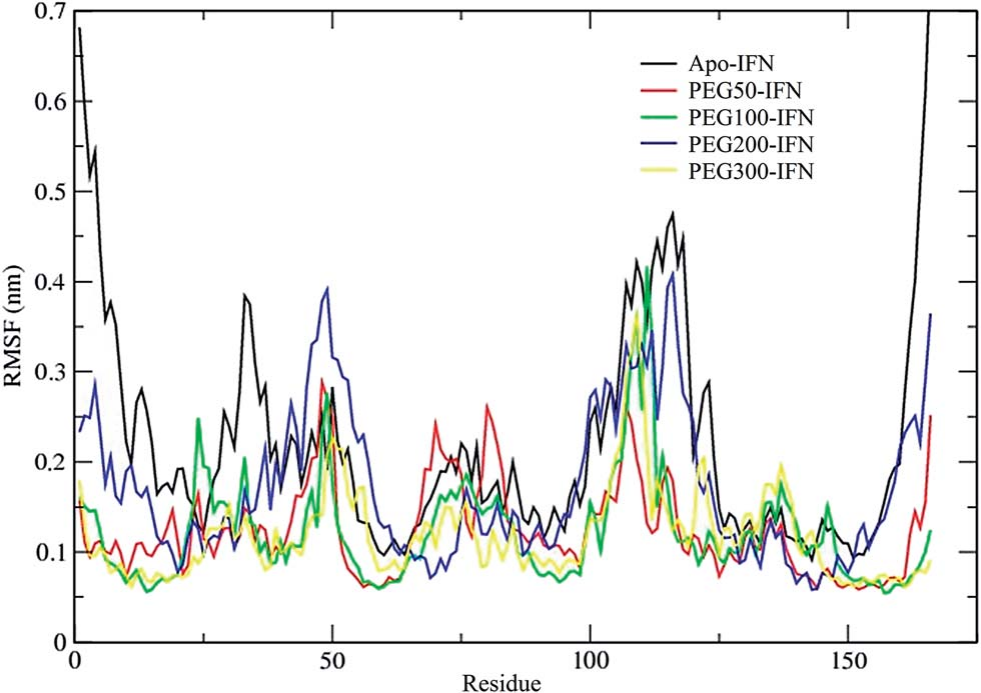
RMSF of apo-IFN and conjugated PEG*x*-IFNs.

The secondary structure of IFN remained unchanged during the simulations for all PEG*x*-IFN conjugated systems. Fig. 3 shows that the 5 α-helices that make up the IFN structure were clearly visible in all simulations. The results confirmed that the covalently bound PEG chains did not affect the native structure of IFN, which is important for IFN to maintain its biological functions. There were occasional transitions between coils, bends, and turns, but they were all transient. Overall, the number of structural transitions were fewer in the conjugated PEG*x*-IFNs than in the apo-IFN, suggesting a stabilizing effect of the PEG chain on the IFN secondary structure. The residue root mean square fluctuations (RMSF) shows the locations and scales of the secondary structure transitions (Fig. 4). Larger fluctuations were found near residues 40, 50, 80, and 120, corresponding to the white and yellow bands (loop/coil/turn segments) found in Fig. 3. All of the PEG*x*-IFN conjugated systems appeared to have smaller magnitude of fluctuations than the apo-IFN, again, indicating a stabilizing effect on the IFN structure.

### 2.4 Effects of PEGylation on IFN bioavailability

**Fig. 5:**
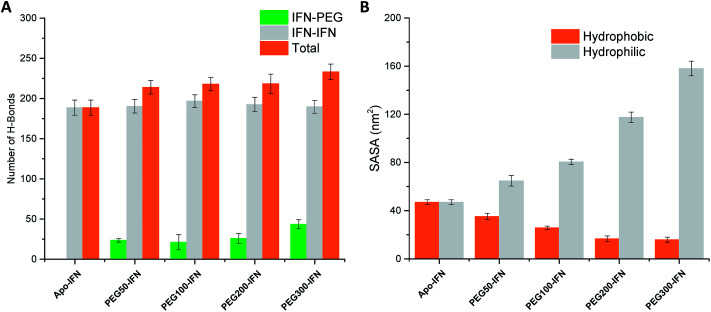
(A) Number of IFN–IFN intramolecular, IFN–PEG intermolecular, and total H-bonds. (B) Hydrophobic (orange) *vs.* hydrophilic (grey) SASA.

Serum half-life or retention time is a critical pharmacokinetic property for protein drugs. It is a measure of how long a drug stays in the circulation before being cleared by the system. Since IFN has poor solubility due to a large number of hydrophobic residues on the protein surface, non-PEGylated IFNs are quickly filtered out from the plasma. PEGs are highly soluble because of its ability to form a large number of H-bonds with water. The attached PEG chains increase IFN solubility by shielding the hydrophobic residues on the protein surface and at the same time forming H-bonds with surrounding water molecules. Fig. 5A shows that the number of IFN intramolecular H-bonds was not significantly altered by the PEGs whereas the number of IFN–PEG intermolecular H-bonds increased substantially, contributing to the shielding effect on the IFN hydrophobic residues.

The effect of PEGylation on IFN solubility can be estimated using solvent accessible surface area (SASA). Fig. 5B shows that as the PEG chain length increases, a large amount of hydrophilic SASA was generated in the PEG*x*-IFN conjugated system while the hydrophobic SASA of IFN was substantially reduced. This drastic modification of SASA and protein hydrophilicity allowed more H-bonds to be formed between the PEGylated IFN and surrounding water molecules, contributing to the increase of IFN solubility. Furthermore, this effect also prevents IFN aggregation. Hydrophobic proteins such as IFNs have a strong tendency to clump together and minimize their contact with the aqueous environment due to the favorable entropy-driven hydrophobic interactions. Shielding the hydrophobic regions of IFN with hydrophilic PEGs reduces the contact between the hydrophobic regions and the chances of establishing hydrophobic interactions, thus effectively resolving the protein aggregation issue.

Another contributing factor is neutralizing antibody (NAb) immunoglobulin G3 binding to IFN, which demolishes IFN bioactivity.^27^ PEF shielding obscures the IFN epitope (residues 1–12 and residues 151–162) recognized by NAbs, effectively blocking NAb binding and contributing to the increased IFN bioavailability and serum half-life.^28^

### 2.5 Effects of PEGylation on IFN bioactivity

**Fig. 6:**
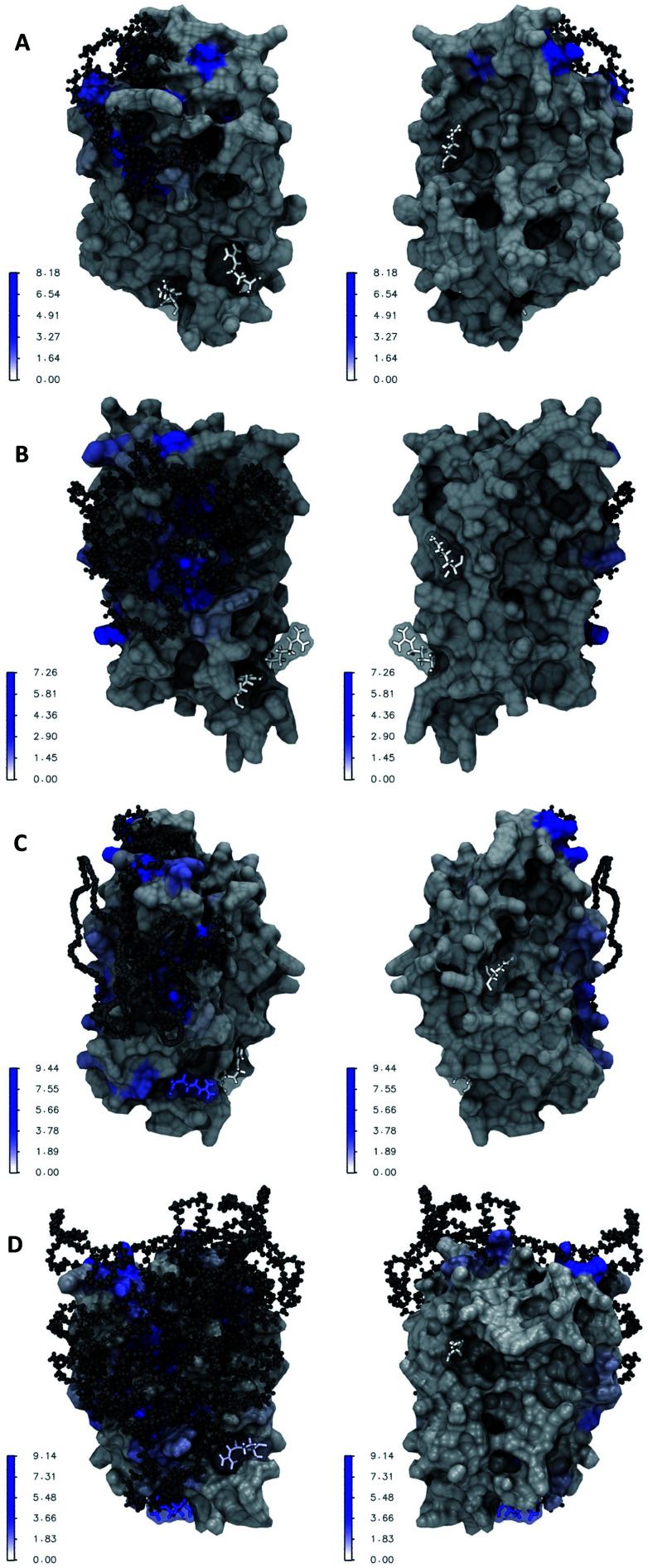
Representative PEG*x*-IFN structures. IFN is shown in the MSMS surface representation with front and back view angles, colored by IFN–PEG contact frequency. PEG is shown in the black CPK representation. Key IFN residues Arg 27, Arg 35, and Lys 123 are shown in the licorice representation with a molecular surface overlay colored by IFN–PEG contact frequency. (A) PEG-50-IFN; (B) PEG-100-IFN; (C) PEG-200-IFN (D) PEG-300-IFN.

Fig. 6A–D illustrate the atomic details of the IFN–PEG interactions. A 4 Å cut-off was used to determine the contact between IFN residues and the PEG chains. The contact frequency was calculated as the average number of contacts between each IFN residue and the PEG chain over the course of the MD simulations. We observed that the PEG chain length increased a larger IFN surface was covered and shielded. Table 2 shows that the residues known to be crucial for antiviral activity, Arg 27, Arg 35, and Lys 123, were not affected in PEG50-IFN and PEG100-IFN. However, Arg 27 was significantly affected in PEG200-IFN and PEG300-IFN. Arg 35 was moderately affected in PEG300-FN. Lys 123 was not affected in PEG200-IFN and PEG300-IFN because of its location at the opposite side of the protein surface. Our data revealed that the benefits of PEGylation are not without limit, and that the intended therapeutic functions of a protein drug could be compromised beyond a certain PEG chain length because of the shielding effect of key functional residues. Indeed, studies have reported that longer PEG chains increase the bioavailability of IFN, but not the therapeutic efficacy of the protein.^15^ In this case, the retention time of a PEGylated IFN system with a 12 kDa PEG chain (equivalent to PEG300-IFN in this study) has been determined to be 29.5 to 34 hours, a 2–3 fold increase over non-PEGylated IFNs.^29^ However, its antiviral activity has been reduced to 66%.^15^ The pharmacokinetic data is consistent with our molecular-level observations in Fig. 6D. In PEG300-IFN, Arg 27 was completely blocked; Arg 35 was partially blocked; and Lys 123 was not blocked at all, allowing the protein to retain a certain level of antiviral properties. Studies have also shown that the antiviral activity of a 20 kDa PEG chain (equivalent to PEG500-IFN) drops to 55% with 4–6 fold increase of retention time over non-PEGylated IFNs. When the PEG chain length is below 200, IFN antiviral activity remains at 100%. Its retention time, however, is merely 10 to 15 hours.^15^ Taken together, the results suggest that there is a tradeoff between increasing retention time and decreasing therapeutic activity. It appears that the optimal PEG chain length with regards to IFN antiviral activity lies between PEG300 and PEG500 (12–20 kDa). Other protein therapeutics have the same issue. It has been reported that PEG500 (20 kDa) is the optimal PEG chain length for the PEGylated human granulocyte colony stimulating factor (GCSF), a protein drug of similar size to IFN, to achieve longer circulation time while retaining bioactivity.^30^

**Table 2:** Contact frequency between PEG and key IFN residues

	Arg 27	Arg 35	Lys 123
PEG50-IFN	0	0	0
PEG100-IFN	0	0	0
PEG200-IFN	3.29 ± 0.05	0	0
PEG300-IFN	3.39 ± 0.01	1.41 ± 0.06	0

## 3. Materials and methods

### 3.1 IFN protein

IFN β-1a (referred as IFN) was used as the representative model system in this study. The crystal structure of IFN was obtained from the RCSB Protein Data Bank (PDB ID: 1AU1).^31^

### 3.2 Building PEG

We focused on linear PEG polymers in this study. The PRODRG online server was used to produce a set of 10 mol2 files for each PEG molecule of length ranging from PEG10 to PEG30.^32^ The AM1-BCC atomic partial charges^33^ for each set of 10 structurally identical PEG molecules were assigned and averaged with antechamber in the AMBER 14 package.^34^ The observed changes of partial charges from short to long PEG chains were used to construct a systematic and self-consistent charge model for assigning atomic partial charges for PEG chains of any length. PEG and propylamine fragments were added to the organic_bld file of Maestro academic version.^35^ PEG–propylamine molecules containing 50, 100, 200 and 300 PEG units were constructed in Maestro.

### 3.3 Linking PEG to IFN

VMD RMSD calculator extension was used to align PEG–propylamines and the IFN N-terminal Met 1 residue on the atoms of the amine group while IFN coordinates were fixed.^36^ The structures were visually inspected to ensure that there were no atomic clashes. Mol2 files containing the new coordinates of the aligned PEG–propylamines were created. VMD molefacture extension was used to remove the amine group of PEG–propylamines, along with one of the amine H atoms in the Met 1 residue. AMBER LEaP was used to create a bond between the N atom of Met 1 and the C1 atom of the PEG–propylamines.^34^ A frcmod file was also created to provide LEaP with the information necessary to recognize the nonstandard residue “PEG” and define the proper dihedral angles and torsions. AMBER LEaP was then used to create the initial files (inpcrd, prmtop, and PDB) for the MD simulations.

### 3.4 MD simulations

MD simulations were carried out using AMBER PMEMD^37^ on free PEG*x*, apo-IFN, and PEG*x*-IFN (*x* = 50, 100, 200, 300) parametrized with the AMBER ff99SB force field^34^ and the custom PEG charge model. Each system was solvated in a TIP3P water box, leaving 10 Å between the solute surface and the box boundary. All crystallographically resolved water molecules were retained. The IFN systems had a net charge of +9, which were neutralized by adding 9 Cl^−^ counterions. An ionic concentration of 0.15 M NaCl was introduced to mimic experimental conditions. Protonation state of the protein residues was determined at pH 7 for the AMBER ff99SB force field by the PDB2PQR web server at http://nbcr-222.ucsd.edu. Simulated annealing procedures were used to effectively optimize the PEGs. With IFN backbone restrained, the systems were gradually heated from 0 K to 500 K at 1 K incremental for every 2 ps, and equilibrated for 200 ps at 300 K, 400 K and 500 K. The systems were then gradually cooled back to 300 K using the same procedure. 25 ns production runs were performed after the simulated annealing process. A time step of 2 fs was used in all simulations. Bonds to hydrogen atoms were constrained using the SHAKE algorithm with a relative geometrical tolerance of 10^−6^. A cutoff of 14 Å was applied to the non-bonded interactions. The output frequency of trajectory files was every 1000 steps (2 ps). The isothermal–isobaric (NPT) ensemble was applied in all simulations with temperature regulation (thermostat) achieved by Langevin dynamics (collision frequency of *γ* = 1.0 ps^−1^) and constant pressure regulated by Berendsen barostat.

To ensure reproducibility, multiple MD simulations were performed for each system. No statistically significant differences were observed among these independent simulations. The MD trajectory of a single representative simulation for each system was analyzed using DSSP,^38^ VMD,^36^ Wordom,^39^ Gromacs,^40^ and in-house scripts. Hydrogen bonds were determined by a donor–acceptor distance of 3.0 Å and a 120-degree angle cutoff. A 4 Å cutoff was used to determine the contact between IFN residues and the PEG chains.

### 3.5 PEG persistence length

To validate our charge model used to parametrize the PEGs and that our MD simulations reflect contact the dynamics of PEGs in physiological conditions, we calculated the persistence length *λ* of the free PEGs from the MD trajectories using the worm-like chain model:^24^1
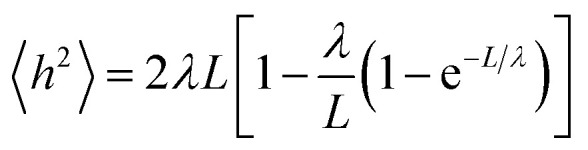
where *h* is the mean-squared end-to-end distance of the PEG series, *L* is the length of the fully extended PEGs; *λ* was calculated by a nonlinear least-squares fit, standard errors of *λ* were estimated by statistical errors of random samples of 〈*h*^2^〉 generated from the MD trajectories.

## 4. Conclusions

Using comparative molecular dynamics simulations, we have provided atomic insights into the improved clinical performance of PEGylated IFNs. We demonstrated the molecular-level evidence related to three areas of IFN clinical performance: (1) PEGylation improves IFN stability by stabilizing protein overall conformation and secondary structures, and restricting residue flexibility without increasing the overall size of the protein drug. (2) PEGylation improves IFN bioavailability and retention time by shielding the hydrophobic residues on IFN surface (reducing protein aggregation), increasing the total number of hydrophilic SASA and H-bonds formed with the aqueous environment (increasing protein solubility), and shielding the epitope residues from NAb binding. (3) However, the benefits of PEGylation come with the cost of reduced IFN bioactivity. Therefore, our conclusion is that protein PEGylation needs to achieve a good balance among the competing benefit and cost factors for the purpose of maximizing the therapeutic potential of the protein drug. With the streamlined computational approach developed in this work, it will be straightforward to study IFN and many other PEGylated protein drugs. Follow-up studies are underway to further investigate the effects of PEGylation sites, PEG structures (linear *vs.* branched), and longer PEG lengths. The structures of PEGylated IFNs obtained from the MD simulations will also provide a good starting point to model the interactions between PEGylated IFNs and IFN receptors, and address the important questions about how PEGylation affects IFN receptor binding at the molecular level.

## Funding sources

This work is supported, in part, by the National Institutes of Health NIGMS IDeA Mountain West Clinical and Translational Infrastructure Network (CTR-IN) Mini and Pilot Grants (1U54GM104944-02/15-746Q-ISU-MG5-00, 5U54GM104944-03/16-746Q-ISU-PG44-00) to D. X., a sub-award from the Institutional Development Awards (IDeA) from the National Institute of General Medical Sciences of the National Institutes of Health (Grants numbers P20GM103408 and P20GM109095) to D. X., the Idaho State University Faculty Seed Grant to D. X., the Idaho State University College of Pharmacy Graduate Student Assistantships to A. T., Y. H, and S. R., the Idaho State University Career Path Interns (CPI) Funding to A. T., and the Boise State University. None of the funders had any role in study design, data collection, data analysis, interpretation, or writing of the report.

## Author contributions

D. X. designed and lead the study; N. S., R. K. S., and S. R. B performed the experiments; N. S., R. K. S., and S. R. B analyzed the data; A. T., Y. H, S. R., and M. L. C contributed to the data analysis and manuscript writing.

## Conflicts of interest

The authors declare no conflict of interest.

## Supplementary Material

RA-008-C7RA12480E-s001
