# Supplementary material for: Molecular insights into the improved clinical performance of PEGylated interferon therapeutics: a molecular dynamics perspective
Source: RSC Adv. 2018 Jan 9;8(5):2315–22. doi: 10.1039/c7ra12480e (PMC9077387; doi:10.1039/c7ra12480e)
Supplement: RA-008-C7RA12480E-s001 [file RA-008-C7RA12480E-s001.pdf]

Electronic Supplementary Material (ESI) for RSC Advances.
This journal is © The Royal Society of Chemistry 2018

Supplemental Information

Principal Component Analysis (PCA) of MD Simulations

The following PCA plots demonstrate that the conformational space sampled in the apo-IFN and PEG-IFN simulations during 3 independent runs is highly consistent and reproducible.

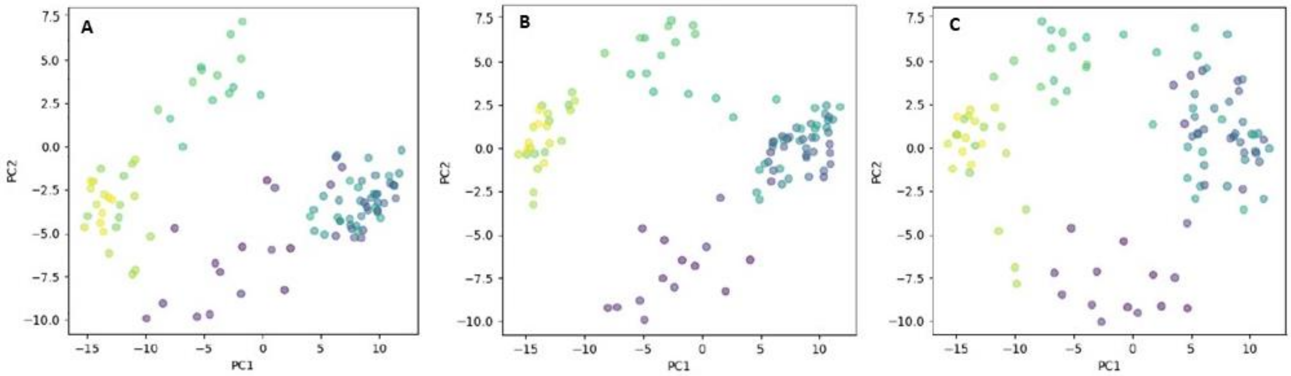

Figure S1. PCA plots of apo-IFN simulations.

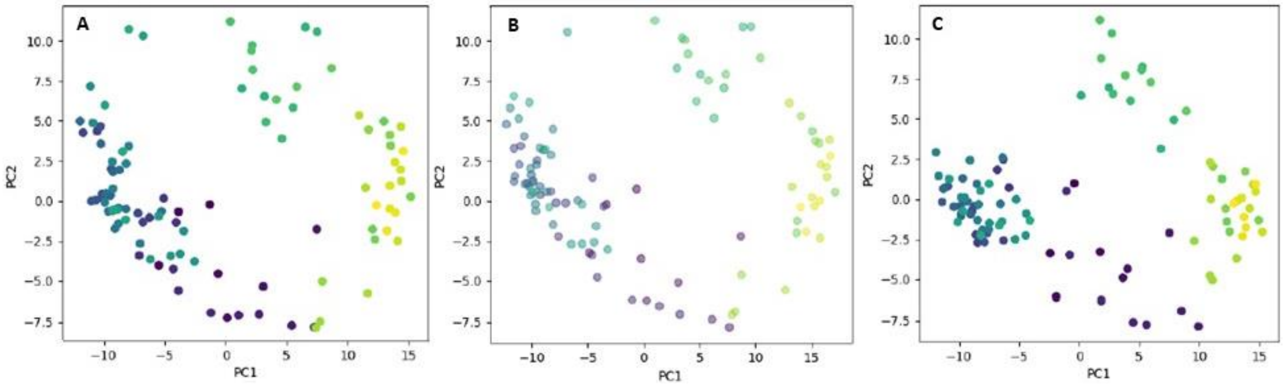

Figure S2. PCA plots of PEG200-IFN simulations.
